# Proto-tool use for food processing in wild Arabian babblers: matching processing methods, substrates and prey types

**DOI:** 10.1007/s10071-024-01866-6

**Published:** 2024-04-24

**Authors:** Yitzchak Ben Mocha, Francesca Frisoni, Oded Keynan, Michael Griesser

**Affiliations:** 1 Department of Biology, University of Konstanz, Universitätsstrasse 10, 78457 Konstanz, Germany; 2 Center for the Advanced Study of Collective Behavior, University of Konstanz, Universitätsstrasse 10, 78457 Konstanz, Germany; 3 Dead Sea and Arava Science Center, Hazeva, Israel; 4 Ben Gurion University of the Negev-Eilat Campus, Eilat, Israel; 5 Department of Collective Behaviour, Max Planck Institute of Animal Behavior, Universitätsstrasse 10, 78457 Konstanz, Germany

**Keywords:** Tool use, *Argya squamiceps*, Birds, Cooperative breeding, *Casama innotata*, *Hyles sp.*, Food extraction, Toxic caterpillars

## Abstract

Cognition is a powerful adaptation, enabling animals to utilise resources that are unavailable without manipulation. Tool use and food processing are examples of using cognition to overcome the protective mechanisms of food resources. Here, we describe and examine the flexibility of proto-tool use (defined as the alteration of an object through object-substrate manipulation) for food processing in a cooperatively breeding bird, the Arabian babbler (*Argya squamiceps*). Field observations demonstrate that the birds transport different caterpillar species to different substrate types depending on the processing method needed to prepare the caterpillar for eating. Species with toxic setae (e.g. *Casama innotata*) are transported to be rubbed on rough substrates (e.g. sand) before consumption, while other species (e.g. *Hyles livornica*) are transported to be pounded against hard substrates until their inner organs are removed and only their external body part is consumed. These results are among the few to describe flexible proto-tool use for food processing in wild animals. They thereby contribute to the taxonomic mapping of proto-tool use and food processing in non-human species, which is a fundamental step to advance comparative studies on the evolution of these behaviours and their underlying cognitive mechanisms.

**Supplementary Information:**

The online version contains supplementary material available at 10.1007/s10071-024-01866-6.

## Introduction

In the course of their evolution, organisms may evolve morphological adaptations that are tailored to overcome specific environmental challenges. For example, the extremely elongated tongs of Wallace's sphinx moths (*Xanthopan praedicta*) enable nectar consumption from the equally extremely elongated spur of Darwin’s comet orchid (*Angraecum sesquipedale*) (Minet et al. 2021). Alternatively, organisms may evolve cognitive abilities enabling them to invent ways to handle novel challenges (Seed and Byrne 2010; Trébouet et al. 2018). Tool use (i.e. where an individual holds a tool and uses it to manipulate the environment: Shumaker et al. 2011), proto-tool use (i.e. where the individual transforms an object through object-substrate manipulation: Parker and Gibson 1977) and food processing are such cognitive and behavioural solutions for new challenges. Testing hypotheses about the evolution of these behaviours requires mapping the taxonomic distribution of tool-assisted food processing and its cognitive complexity in various species (Lefebvre et al. 2002).

Taxonomically, the use of tools and proto-tools to extract and process food has been documented in fish, birds and mammals (Bentley-Condit and Smith 2010; Shumaker et al. 2011). For example, tool use has been observed in Egyptian vultures (*Neophron percnopterus*) and bristle-thighed curlews (*Numenius tahitiens*) who throw stones to crack thick-shelled eggs (i.e. the stone serves as a tool) (van Lawick-Goodall and van Lawick-Goodall 1966; Marks and Hall 1992). Proto-tool use was observed, for instance, in a red-tailed hawk (*Buteo jamaicensis*) and sea otters (*Enhydra lutris*) who kill snakes and crack mussels, respectively, by hitting them against a boulder (i.e. the boulder serves as a proto-tool) (Ellis and Brunson 1993; Haslam et al. 2019).

However, in relatively few species the flexibility involved in tool-assisted food processing has been studied in detail. Maybe the most extensively studied example are New Caledonian crows (*Corvus moneduloides*) that demonstrate extreme flexibility in tool preparation, usage and cultural differences (Bluff et al. 2010; Auersperg et al. 2011; Klump et al. 2019). Other examples of tool-assisted food processing that were studied in detail are glaucous-winged gulls (*Larus glaucescens*) who match their clams cracking methods according to the probability of the food being stolen by conspecifics (throwing it against a substrate while standing versus flying) (Barash et al. 1975) and Western gulls (*Larus occidentalis*) who drop shells from different heights according to the weight of the shell (Maron 1982). The paucity of in-depth studies of tool-assisted food processing across many species hinders our understanding of the mechanisms that underlie these behaviours (Seed and Byrne 2010). For example, most species in animal tool use catalogues are reported to use tools for only one out of ten purpose types (e.g. food preparation, mate attraction; Bentley-Condit and Smith 2010; Shumaker et al. 2011). However, since most catalogues' entries are based on anecdotical observations (e.g. Sedgwick 1951; Wellenstein 1986; Coyer 1995; Drennen 1995), it is not clear whether this reflects the cognitive inflexibility of animal tool use or an artefact of the scarcity of detailed observations within species.

Here, we describe the use of proto-tools for food processing and food extraction in the Arabian babbler (*Argya squamiceps*) and investigate the flexibility of these behaviours to increase detailed knowledge on animal tool use across species. Arabian babblers are cooperatively breeding birds living in the deserts of the Arabian and Sini Peninsulas, Palestine and Israel (IUCN 2016). These medium-sized songbirds reside year-round in stable social groups of 2–20 members of both sexes and diverse ages (Zahavi 1989; Keynan and Ridley 2016). All group members provide proactive alloparental care to offspring in the group (Ostreiher 1997; Ben Mocha et al. 2019). Arabian babblers are omnivorous birds, whose diet is mostly composed of a variety of arthropods (e.g. solifugae, caterpillars, butterflies, termites; video S1) with some vegetative items such as flowers, nectar (e.g. *Loranthus acacia*) and fruits (e.g. *Nitraria retusa* and *Lycium shawii*). Though, living in an extreme desert with unpredictable rainfall (mean annual rainfall at our study site: 45 ± 26 mm; Israel Meteorological Services) makes the diet composition of Arabian babblers highly variable across seasons.

In terms of tool use, Arabian babblers have been shown to present objects to invite conspecifics for copulation (Zahavi 1988; Ben Mocha and Pika 2019) and play (Pozis-Francois et al. 2004). These object presentations are qualified as tool use given that they involve the external employment of an unattached object to alter the condition of another organism by holding the tool (see tool use for mate attraction in Bentley-Condit and Smith 2010; Shumaker et al. 2011). However, virtually nothing is known about whether Arabian babblers utilise environmental objects for foraging.

The aim of the current study is twofold. First, we describe two proto-tool behaviours used by Arabian babblers for food processing and food extraction: “rubbing” (videos S2–4) and “pounding” (video S5) of prey against different substrate types. Second, to examine the flexibility of these behaviours, we investigate whether Arabian babblers match prey types (caterpillars with different protective mechanisms) with food processing methods (rubbing/pounding) and the processing substrate (rough/hard).

## Methods

### Study site and population

We observed a wild population of Arabian babblers at the Shezaf Nature Reserve (30.718N, 35.266E) and its surrounding areas in Israel. Birds are marked with a unique combination of coloured plastic rings and habituated to human presence to the degree that observations can be made at a distance of 2–20 m. Continuous monitoring of the population since 1971 provides comprehensive knowledge of the life history, sex and dominance rank of most individuals (Zahavi 1989; Dragić et al. 2022).

### Datasets

The current study is based on two data sources. First, in May–June 2014 and December 2023, we systematically observed the foraging behaviour of 16 individuals from four social groups for a maximum of 20 min each (mean ± SD observation length per individual = 16.2 ± 5.9 min). Data recording was paused when the focal bird stopped foraging (e.g. to perform sentinel behaviour or allopreening) and started again when foraging was resumed. Data were recorded on a smartphone with the CyberTracker software (CyberTracker, 2015) and/or filmed with a digital high-definition video camera (Canon LEGRIA HFM41) while simultaneously narrating the observed behaviour.

Second, we searched in our Arabian Babbler Behavioural Media Library for recordings of foraging behaviour. This library consists of video clips recorded in the study population during January-June 2010, August 2011-July 2012, February-June 2014 and December 2023. Videos were either recorded opportunistically when interesting behaviours were observed or during systematic data collection for other research purposes (e.g. Ben Mocha 2013; Ben Mocha et al. 2019). The search of this Media Library yielded 37 recordings depicting the acquisition of 109 food items by 20 individuals from seven social groups.

### Data coding

Windows Media Player and Excel were used to code the video recordings. For each food item obtained by the focal individual, the following variables were coded: (1) ID, social group, sex and age (in months) of the focal individual. (2) Categorical age of the focal individual (younger or older than 15 months). We distinguish between younger and older than 15-month-old birds because Arabian babblers are independent of alloparental feeding at about three months of age (Ridley 2007). Hence, birds that are older than 15 months had been feeding independently for over a year, and thus, had been exposed as independent foragers to the variety of food types available in their habitat throughout the annual cycle. (3) Recording modus (systematic or opportunistic observation of foraging). (4) Food category (pupa inside a cocoon/caterpillar/insect/fruit/other/unknown). We note that more accurate categories were used when possible (e.g. caterpillar with setae, ant, termite, butterfly, *Casama innotata*; see ESM for an account on prey type observed), but given that prey were consumed, identification at the species level is based on the analysis of video recordings and should be taken with caution. (5) Whether the food was consumed in the location it was found or was transported to another location (not transported/transported). (6) The type of substrate on which the food item was found (vegetation [e.g. a bush, tree trunk]/rough [e.g. sand, ground covered with dry branches]/hard [e.g. solid and bare ground, a solid dead trunk]). (7) The type of substrate on which the food item was processed or consumed if it was not processed (same categories as (6)). (8) Whether the food was processed before consumption (yes/no). (9) If the food was processed, how it was processed (rubbed against the substrate/pounded against the substrate/both actions). Following (Shumaker et al. 2011, Table 1.1) “rubbing” was defined as moving “an object across a bodily surface, often repeatedly, while applying pressure” and “pounding” as hitting “an object or prey item forcefully, often repeatedly, with a second relatively hard object (the tool)”. Note that, in our study, it was the prey (i.e. the object of change) that was rubbed or pounded against the agent of change (i.e. the substrate) and not the tool that was rubbed or pounded against the prey. (10) Whether the food finder ate the food or delivered it to another individual. (11) The duration of processing (in seconds) from the moment the bird started transporting or processing the food until it started eating it (or moved to deliver the food to another bird). (12) Which part of the food was consumed (the entire prey/only the external part of the prey/only the pupa extracted out of the cocoon). Since not all parameters were available for all episodes, different sample sizes are reported for different analyses.

### Statistical analyses

Statistical analyses were conducted using RStudio (R version: 4.2.2). All tests were two-tailed and the significance level was set to α = 0.05. Three Generalized Linear Mixed Models (GLMM) (Baayen 2008) were used to account for the non-independence of data caused by repeated observations on the same individuals (Waller et al. 2013). The actor ID was included as a random intercept effect in all models. Models were fitted with the functions glmer in the lme4 R package (version 1.1.35.1) (Bates et al. 2015), using a binomial error structure and logit link function (McCullagh and Nelder 1989; Baayen 2008). The significance of each fixed-effect predictor was determined by comparing the full model with a reduced model lacking each one of the fixed-effect predictors at a time (R function”drop1″ with argument test “Chisq”: Schielzeth and Forstmeier 2009). The overall significance of each GLMM was tested by comparing the model with its corresponding null model (containing only the intercept and random effects) using a likelihood ratio test. Models stability was assessed by excluding levels of random effects one at a time from the dataset and comparing model estimates derived from these data with those derived from the full dataset. The normality of residuals and the absence of outliers, under-dispersion and over-dispersion was confirmed using the R package DHARMa (version 0.4.6) (Hartig 2022).

The first GLMM examined the relationship between the substrate type on which a prey item was processed and the method used to process it. The response variable was the processing method (rubbing/pounding the prey against the substrate). The model had two fixed effect predictors: (i) the type of substrate on which the prey item was processed (rough/hard), and (ii) actor categorical age (younger/older than 15 months old). Two prey items that were processed by rubbing and pounding were excluded from the analysis.

The second GLMM tested whether food items that required processing were more likely to be transported to a new location (i.e. to the processing substrate) before consumption than food items that were not processed. The response variable was whether the food was transported (yes/no). The model had two fixed effect predictors: (i) whether the food item was processed before consumption (yes/no), and (ii) actor categorical age (younger/older than 15 months old). Food items that were fed to another bird were excluded as they may have been transported to feed the other bird.

The third GLMM focused on food items that were transported from where they were found. The response variable was whether the substrate type to which the food was transported was of different/same type than the substrate type on which the food was found (substrates were coded as either rough or hard type). The model had two fixed effect predictors: (i) whether the food was processed before consumption (yes/no), and (ii) actor categorical age (younger/older than 15 months old). Food items that were used to feed another bird were excluded as they may have been transported to feed the other bird. Due to too few repetitions per bird ID (the random effect), this third model did not converge well. We thus also repeated this analysis with a Fisher Exact Test to test the association between the response variable and the main fixed effect in the model (whether the food was processed or not). Similar to the null-full model comparison of the GLMM test, the Fisher Exact Test yielded a significant result (two-tailed p = 0.003).

Testing each of these three models using log-transformed (to enable normal distribution) age in months as a continuous variable resulted in similar results to using age as a categorical variable.

## Results

### Arabian babblers use proto-tools to process food

**Fig. 1:**
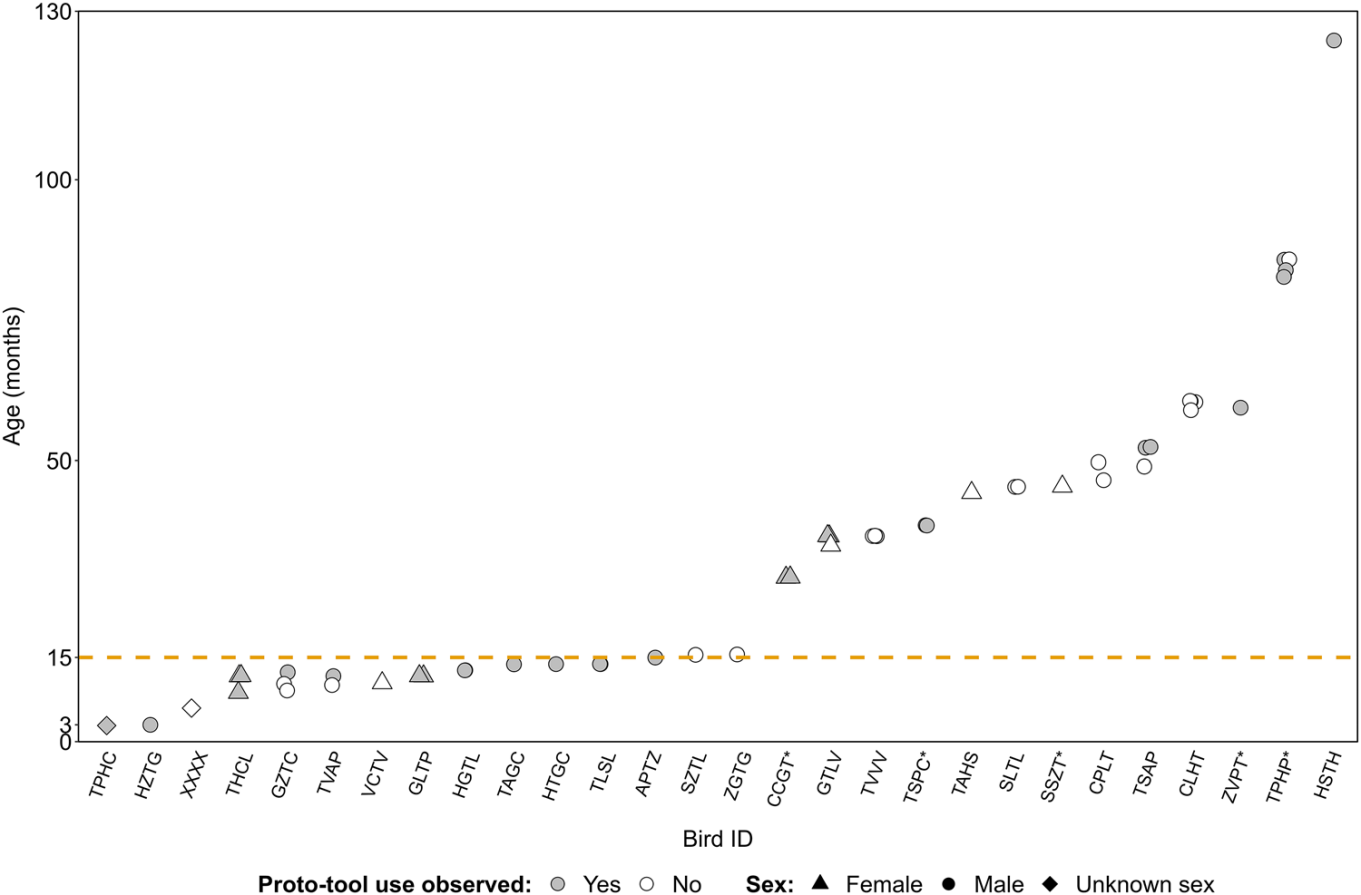
Age and sex distributions of the observed birds according to whether they used proto-tool for food processing at a specific age**.** The orange dashed line distinguishes between birds younger and older than 15 months old. The five birds marked with asterisk joined the research population as adults (i.e. > 12 months old) and their minimum age at the time of observation is depicted

The foraging behaviour of 28 birds (19 males and 7 females, 2 birds with unknown sex) from seven social groups was observed in April 2010, February-June 2014 and December 2023. The age of these birds ranged from three months to ten years. Out of this sample, 20 birds from five groups were observed using a substrate to process and/or extract food. Birds from both sexes (15 males and 4 females) and both age categories (11/9 birds younger/older than 15 months old, respectively) processed food (Fig. 1).

A total of 236 food items were observed being consumed. Out of 186 items identified as prey, 42% were processed on a substrate before consumption. However, the natural frequency of proto-tool food processing is likely more similar to its rate observed during the systematic observations on foraging (26% out of 109 prey consumed). Fruits and flowers were consumed without processing (N = 11 items).

On four occasions, birds pinned the prey to the substrate with one leg and used their beak to tear the prey into edible and inedible parts. These food processing cases were excluded from our analyses of proto-tool food processing.

### Processing methods

Prey were processed by holding it with the beak and rubbing (N = 54, videos S2-S4) or pounding (N = 22, video S5) or rubbing and pounding (N = 2) it against a substrate.

The main function of rubbing the prey seems to be the removal of toxic secretions. For example, most prey processed by rubbing or by rubbing and pounding (31 out of 56) were caterpillars with long setae. We identified these caterpillars as *Casama innotata* (Video S2-S3), a species with long setae assumed to be poisonous (Laajimi et al. 2020). Overall, *Casama innotata* caterpillars were never consumed unprocessed (100%, N = 32), almost all of them were processed by rubbing (97%, N = 31) and were often consumed entirely (87%, N = 15). In only two cases *Casama innotata* caterpillars were rubbed against a substrate before the bird used its beak and leg to extract the caterpillar’s inner parts and ingested only its outer body part. All five caterpillars with setae that were used for allofeeding (Kalishov et al. 2005; Ben Mocha and Pika 2019), were first processed by the prey finder before feeding it to the other bird. Other prey processed by rubbing were small insects (e.g. ants) and cocoons (see below and ESM).

Birds pounded the prey against the substrate to extract its internal organs (Video S5). All caterpillars processed by pounding were pounded until their inner parts (intestines) did spill out and only the external body part was consumed (N = 11). Almost all caterpillars processed by pounding did not have setae (16 out of 17) and some of them were identified as *Hyles livornica* (Video S5). Other prey processed by pounding were three small insects and two pupas in cocoons (see below).

Cocoons were rubbed (N = 14), pounded (N = 2) or rubbed and pounded (N = 1) until the pupa was extracted (Video S4). Pupas were subsequently eaten entirely while the cocoons were not consumed (N = 7).

The mean ± SD length of food processing was 37.5 ± 49.3 s (N = 26 events, duration values are underestimated as most video recordings started shortly after the bird started transporting the prey).

### Processing methods and substrates are selected to match each other

**Fig. 2:**
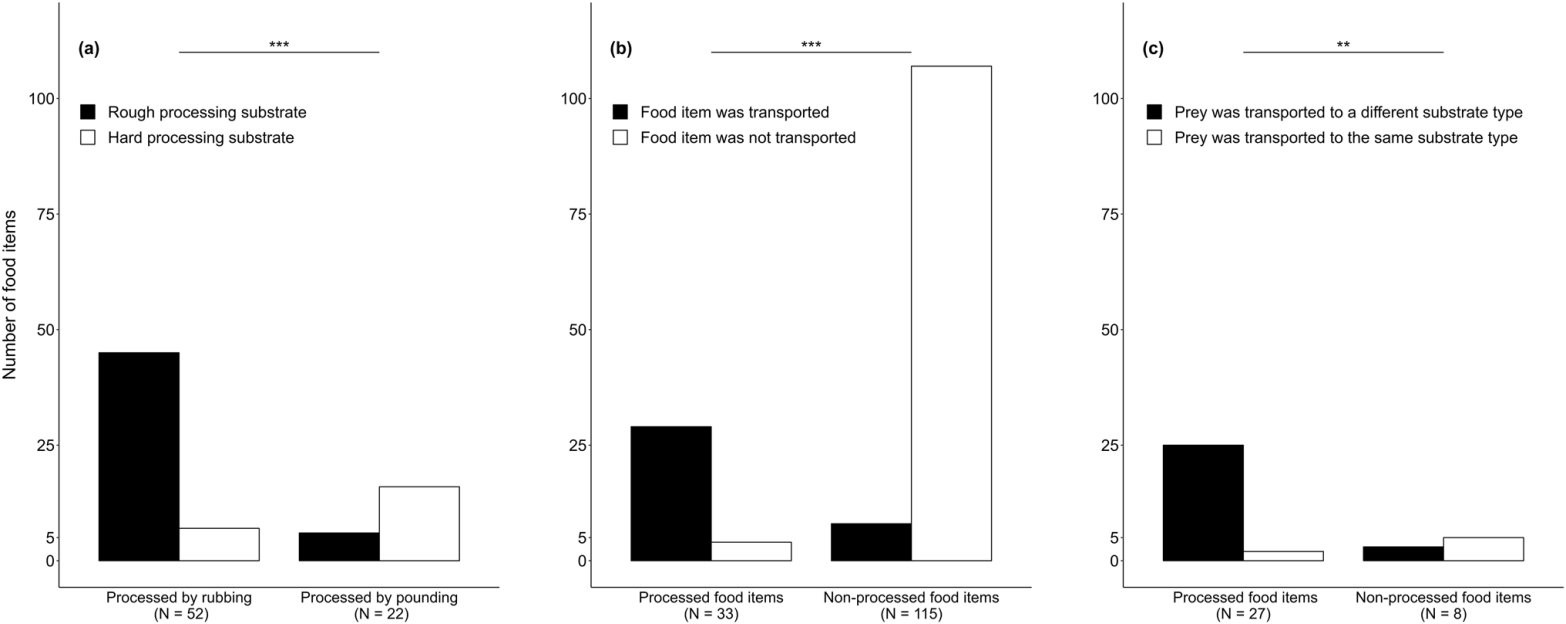
**a** Number of prey items according to the processing method (rubbing/pounding) and the substrate they were processed against (rough [e.g. sand]/hard [solid ground], total N = 74). **b** Number of food items according to whether they were processed (yes/no) and whether they were transported to a new location or consumed where they were found (total N = 148). Food items that were delivered to another bird were excluded. **c** Number of food items that were transported according to whether they were processed (yes/no) and whether the substrate they were processed on was of the same or different type than the substrate they found on (n = 35). Food items that were delivered to another bird were excluded

Prey processed by rubbing were more likely rubbed against a rough substrate (e.g. sand; 87% of the 52 prey items processed by rubbing) than a hard substrate (e.g. solid ground or a nearby stick). In contrast, prey items that were processed by pounding were more likely pounded against a hard substrate than a rough one (73% of the 22 prey items processed by pounding; full-null model comparison: χ^2^ = 20.387, d.f. = 2, P < 0.001; Fig. 2a and Table 1).

**Table 1:**
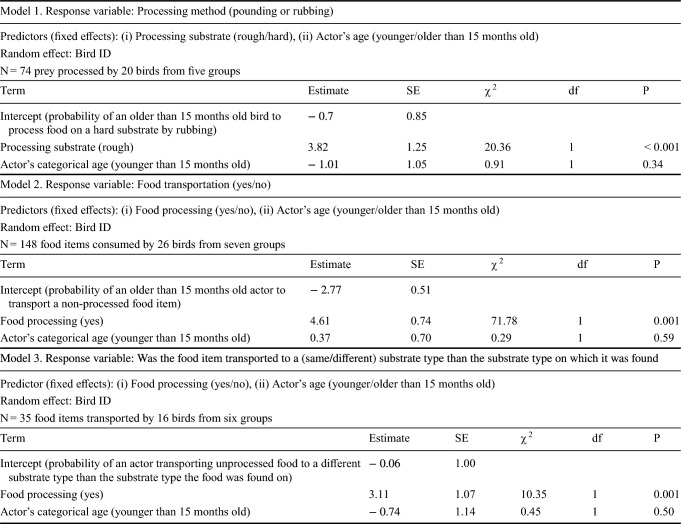
Results of Generalized Linear Mixed Models

Food items that required processing were more likely transported from where they were found to a new location where they were processed and consumed (88% of 33 processed food items, videos S2 and S4). While non-processed food items were usually consumed where they were found (93% of 115 non-processed food items, video S1; full-null model comparison: χ^2^ = 75.238, d.f. = 2, P = 0.001; Fig. 2b and Table 1).

Considering only food items that have been transported, items that required processing were more likely transported to a different type of substrate than the substrate where they were found (93% of the 27 food items) than food items that were not processed (38% of the 8 food items; full-null model comparison: χ^2^ = 10.633, d.f. = 2, P = 0.005; Fig. 2c and Table 1). Accordingly, prey that were processed by rubbing were usually transported to a rough substrate (total N = 12; Table 2a). In contrast, prey that were processed by pounding were mostly transported to a hard substrate (total N = 14; Table 2b).

**Table 2:** Processed prey items according to the type of substrate they were found on and the type of substrate they were transported to be processed on

	Type of substrate the prey was transported to be processed on:	
	Rough	Hard
Type of substrate the prey was found on:		
(a) Prey processed by rubbing (N = 12)		
Vegetation (e.g. a tree trunk, a bush)	8	2
Rough (e.g. sand)	1	0
Hard (e.g. solid ground)	1	0
(b) prey processed by pounding (N = 14)		
Vegetation (e.g. a tree trunk, a bush)	2	6
Rough (e.g. sand)	1	5
Hard (e.g. solid ground)	–	–

## Discussion

The present study provides the first description of proto-tool use for food extraction and processing in the Arabian babbler population at the Shezaf Nature Reserve, Israel.

**Fig. 3:**
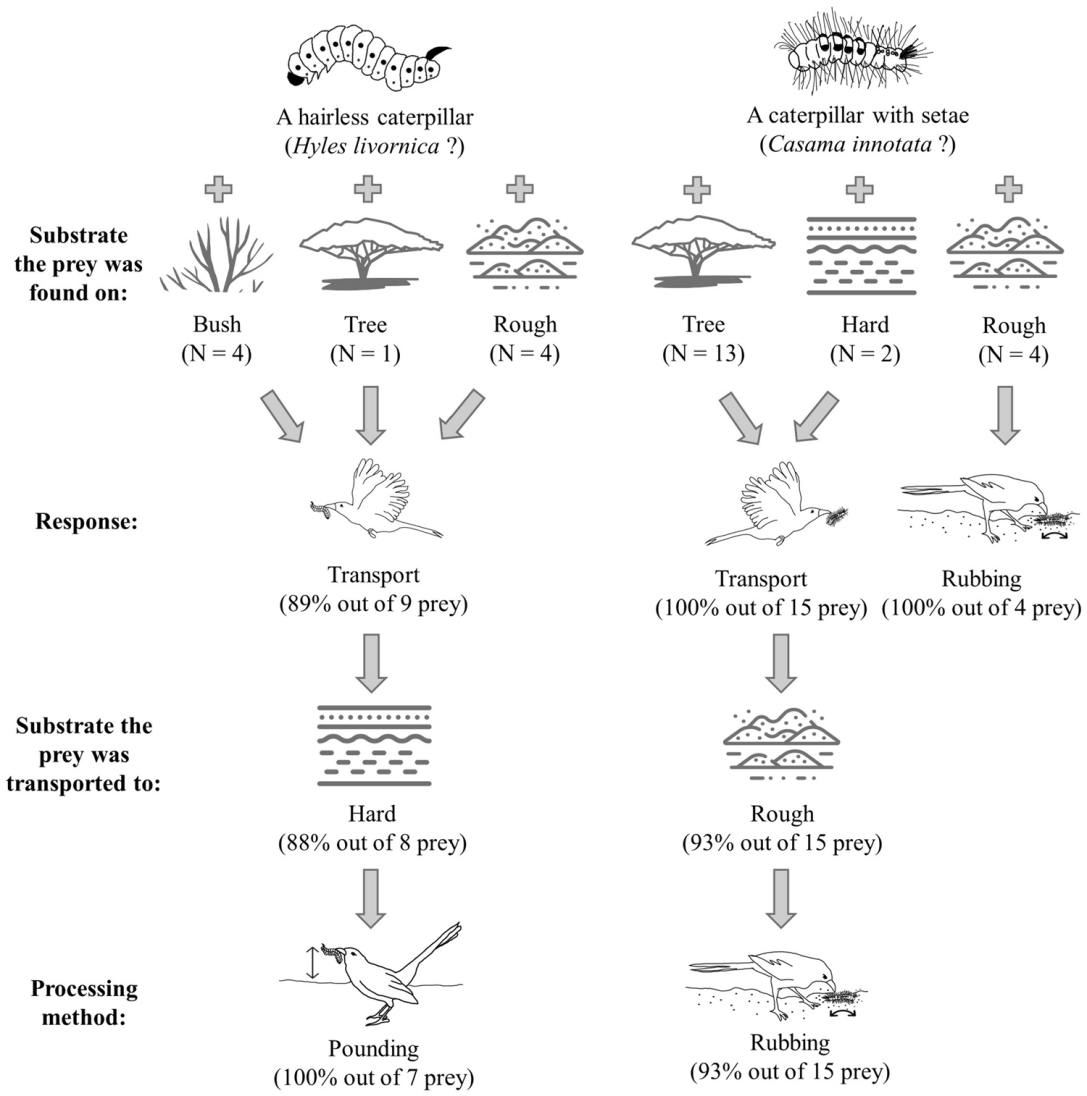
A decision-making flowchart of the matching between prey, types of processing substrate and processing methods at the Shezaf Nature Reserve population of Arabian babblers, Israel. Caterpillars and babblers icons by Francesca Frisoni; hard soil icon by tezar tantular (thenounproject.com); bush icon by TRAVIS BIRD (thenounproject.com); tree icon by Isabelle Ryan (thenounproject.com); rough sand icon by Freepik (flaticon.com)

In this population, Arabian babblers use three forms of proto-tool food extraction and processing (Fig. 3). First, to extract pupas out of their cocoons, the cocoons are pounded and/or rubbed against a rough substrate (usually ground covered with dry pieces of acacia seed pods; video S4). Second, preys with potentially toxic setae (e.g. *Casama innotata*: Laajimi et al. 2020) are carefully rubbed against a rough substrate (often sand) before the entire caterpillar is eaten (videos S2-S3). Third, some caterpillar species that lack setae (e.g. *Hyles livornica*) are pounded against a hard substrate until their intestines spill out and only the external body part is consumed. The midgut of *Hyles livornica* caterpillars may contain unprocessed toxins (Mebs et al. 2017; Hundsdoerfer et al. 2019). In addition, over 80% of *Hyles livornica* caterpillars found in the study region were parasitized by Tachinidae flies (Katbeh-bader 2014). Accordingly, Arabian babblers may remove the intestines of these caterpillars because they contain unprocessed toxins or are infected by endoparasites.

Further research is needed to clarify why Arabian babblers process specific caterpillar species. Extensive effort is invested in processing while only the external part of some caterpillars is consumed (Video S5). The nutritional value of these caterpillars should thus be examined in comparison to other prey that co-occur in time and space to test whether they are “fallback” food during poor periods (Trébouet et al. 2018) or whether they have a high caloric value or unique nutrients (Siegfried 1977; Maron 1982). In addition, systematic observations of diet composition across the different seasons are required to assess the ecological significance of proto-tool use for Arabian babblers’ diet.

Arabian babblers extract and process prey by holding it in their beak and manipulating it against a substrate. According to restrictive definitions that require tool users to hold a detached agent of change (i.e. the tool; Shumaker et al. 2011), these behaviours are not qualified as tool use since the bird holds the *object* of change (i.e. the prey) and not the *agent* of change (i.e. the substrate). Rather, the behaviours of Arabian babblers are qualified as proto-tool use, defined as actions “involving the transformation of an object through object-substrate manipulation” (Parker and Gibson 1977, p. 625). Hence, in addition to the already described tool manufacturing (Ben Mocha and Pika 2019) and tool use to invite conspecifics for copulation (Zahavi 1988; Ben Mocha and Pika 2019) and play (Pozis-Francois et al. 2004), the current study expands the known tool kit (all various tools known to be used by the species: Shumaker et al. 2011) of Arabian babblers to include proto-tool use for food extraction and food processing.

Multiple authors emphasized the importance of behavioural flexibility to infer more cognitively demanding tool use (St Amant and Horton 2008; Raby and Clayton 2009; Seed and Byrne 2010; Ben Mocha and Burkart 2021). Our results show that Arabian babblers use different solutions to overcome different protective mechanisms by adequately matching different prey types, processing methods and processing substrates (Fig. 3). This flexibility may suggest some understanding of the processing required to overcome different protective mechanisms (Trébouet et al. 2018; Ben Mocha and Burkart 2021), and thereby, further support the hypothesis that the distinction between proto-tool use and tool use is semantic rather than entailing cognitive difference (Shumaker et al. 2011). Future observational and experimental studies should investigate the cognitive complexity involved in the tool use of Arabian babblers.

On a more general level, our results propose a role of extended parenting in terms of parenting period and number of alloparents for the emergence of individually and socially learned skills such as proto-tool use and food processing (Hrdy 2007; Uomini et al. 2020). Arabian babblers exhibit social learning abilities (Keynan et al. 2015, 2016) and live in cohesive social groups that provide daily opportunities for such learning (Ben Mocha et al. 2018; Dragić et al. 2021). Future research should, therefore, examine whether the food-processing behaviours of Arabian babblers are genetically hardwired, individually learned or transmitted culturally (Whiten et al. 1999; Galef and Laland 2005). In particular, by focusing on the development of foraging skills in younger than 15 months old birds (e.g. gulls: Barash et al. 1975; Siegfried 1977; New Caledonian crows: Bluff et al. 2010) and between populations differences (Whiten et al. 1999; Kühl et al. 2019).

## Supplementary Information

Below is the link to the electronic supplementary material.

Supplementary file1 (XLSX 35 KB)

Supplementary file2 (MP4 598526 KB)

## Data Availability

The data used for this study is available as Electronic Supplementary Materials.
